# Binocular visual function after staged implantation of extended-depth-of-focus intraocular lens targeting emmetropia and -0.5 diopter: A prospective comparison

**DOI:** 10.1371/journal.pone.0238135

**Published:** 2020-08-25

**Authors:** Yuka Ota, Hiroko Bissen-Miyajima, Kunihiko Nakamura, Manabu Hirasawa, Keiichiro Minami

**Affiliations:** Department of Ophthalmology, Tokyo Dental College Suidobashi Hospital, Tokyo, Japan; National Taiwan University Hospital, TAIWAN

## Abstract

The prospective comparative case series aimed to evaluate the binocular uncorrected visual acuities (BUCVAs) after staged implantations of extended-depth-of-focus intraocular lenses (EDOF IOLs) targeting emmetropia and -0.5 diopter (D). Diffractive EDOF IOLs with an add power of +1.75 D were implanted in the first eyes targeting emmetropia or -0.5 D according to the patients’ preferences, then the targets for the second eyes were determined 1 week or longer after the implantation. IOL powers were determined with the SRK/T formula. Consequently, the subjects were divided into 3 groups: those with emmetropia targeted bilaterally (group EE, 22 patients), those with -0.5 D targeted bilaterally (group MM, 21 patients), and those with monovision of emmetropia and -0.5 D (group EM, 21 patients). Manifest refraction spherical equivalent (MRSE), BUCVA from 0.3 to 5 meters, spectacle use, and questionnaire regarding photic symptoms and patient satisfaction were assessed 3 months postoperatively. No significant differences were seen in the mean BUCVAs at any distance (P > 0.23), spectacle use (P = 0.13), or photic symptoms and patient satisfaction (P>0.65). When the EE and MM groups were assigned based on the MRSE, the EE group was better at 5 m (P = 0.005) while the MM group at 0.5 m (P = 0.031). The effect of different targeted refractions was not identified due to insufficient accuracy in the use of the SRK/T power calculation.

## Introduction

Multifocal intraocular lenses (IOLs) with two foci are used to correct presbyopia in patients undergoing cataract surgery. Resultant good visual acuities at far and near distances have been achieved and postoperative spectacle dependence decreased [1–3]. However, the risks of photic phenomena inherent in the use of bifocal optics are concerns. Extended depth-of-focus (EDOF) is an alternative concept of presbyopia-correcting IOLs. The continuous extension of the depth of focus in distance vision toward intermediate distances can be obtained, and no secondary focus minimizes photic phenomena [4]. A diffractive EDOF IOL with an echelle grating is available commercially. The first-order diffraction constitutes the focus for far distance vision, and the second-order diffraction produces a focus of +1.75 diopters (D) higher than the far focus. The two foci of the small refractive differences consequently prolong the range of vision from far to intermediate distances. Thus, the visual outcomes at a near distance such as 0.3 m are fundamentally lower than with multifocal IOLs with a 4.0-D add power. To compensate for insufficient near vision, monovision has been considered with the targeted refractions of emmetropia and slight myopia [5–7]. A multicenter prospective case series of 411 patients demonstrated that monovision effectively improved the binocular near visual acuities compared with binocular emmetropia [5]. Although the defocus curve indicates the potential for a longer range by adjusting the targeted refraction [8], the effect of myopic targeting has not been assessed. The current prospective comparative case series evaluated the binocular visual outcomes after staged implantation of diffractive EDOF IOLs for three targeted refractions, i.e., bilateral emmetropia, bilateral slight myopia, and monovision of the two refractions.

## Patients and methods

### Participants

The institutional review board of Tokyo Dental Collage (identifier, 800) approved the study protocol. All patients provided written informed consent after explanation of the study, and the study was conducted according to the tenets of the Declaration of Helsinki. Patients scheduled to undergo cataract surgery with bilateral EDOF IOL implantation were recruited. Eye for which the postoperative astigmatism would be anticipated to be over 1.25 D were excluded. The exclusion criteria also included a previous ocular surgery; chronic or recurrent uveitis; acute ocular disease or external/internal infection; diabetes with retinal changes; glaucoma; exfoliation syndrome; pathological miosis; keratoconus; corneal endothelial dystrophy; and capsular, zonular, and pupillary abnormalities.

### Implanted EDOF IOLs

All patients underwent cataract removal and implantation of a one-piece, violet light blocking, hydrophobic acrylic, diffractive EDOF IOLs with an add power of +1.75 D (model ZXR00V, Johnson & Johnson Surgical Vision, Santa Ana, CA). The diffractive optics was designed for providing visual acuity in the range of 0.7 meter to far distances [4,9]. The IOL optics of 6.0-mm diameter had aspheric design on the front surface, continuous sharp optic edges on the posterior, and anteriorly shifted haptics. As the IOL was not optimized for the Barrett Universal II formula (http://calc.apacrs.org/barrett_universal2105/), the IOL power was determined using the SRK/T formula with axial length and keratometry values measured using the IOLMaster 700 (Carl Zeiss Meditec, Inc., Dublin, CA).

### Staged implantation

At first, the eye with advanced lens opacity or lower visual acuity was operated. Therefore, the first eyes included both dominant and non-dominant eyes. After the cataracts were removed using a LenSx^®^ Laser System (Version 2.23) and a Centurion Vision System (Alcon Laboratories, Fort Worth, TX) by a phacoemulsification and aspiration technique [10], the IOLs were implanted completely within the capsules using the IOL inserter system. The targeted refraction for the first eyes was either emmetropia or -0.5 D based on patient preference. With the target of a -0.5-D refraction, the better visual acuity around 50 centimeters was anticipated [5–7].

**Fig 1:**
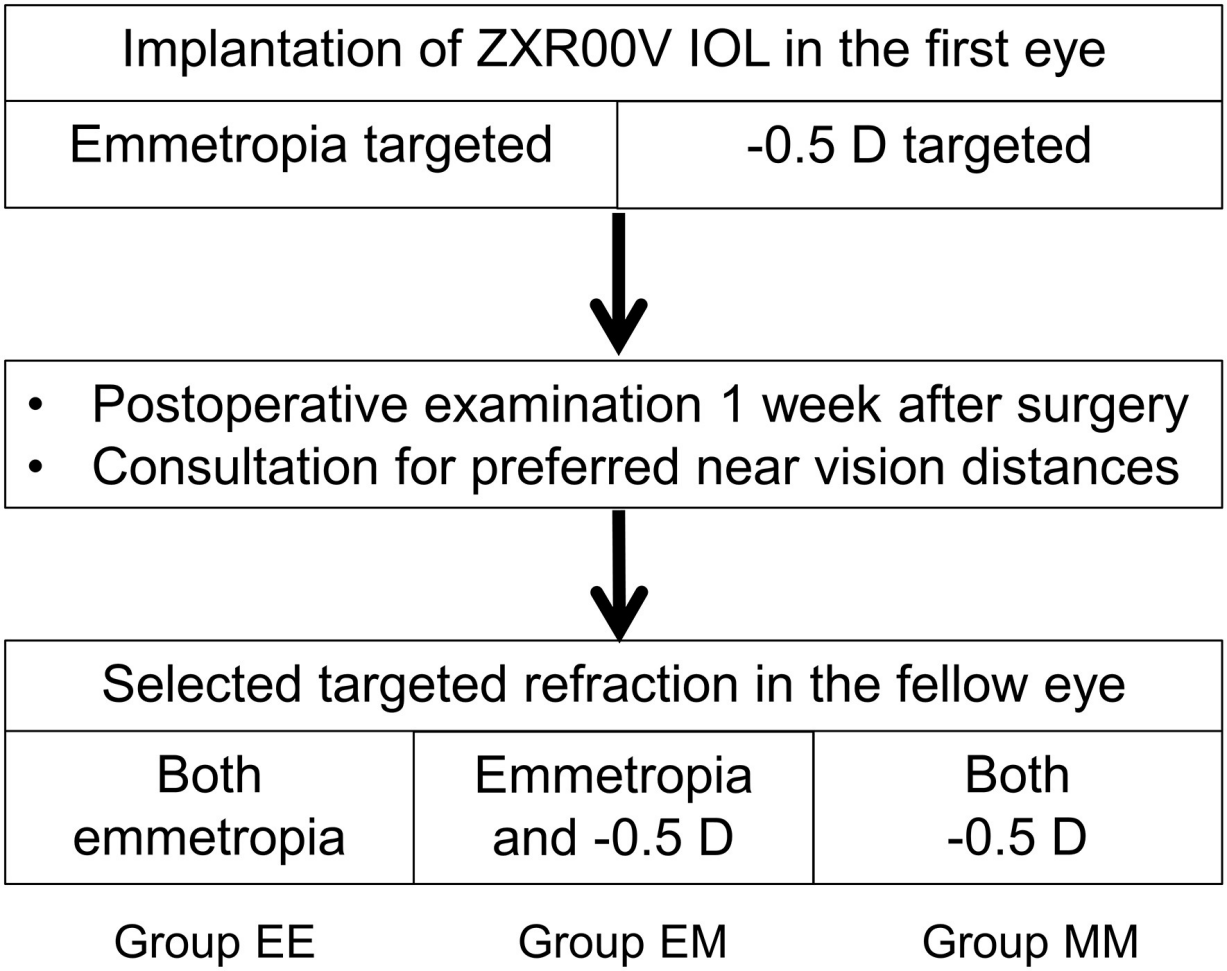
Staged implantation of an extended-depth-of-focus intraocular lens (IOL, ZXR00V) targeting emmetropia and -0.5 diopter (D). The patients are divided into three groups based on the choice of the target refractions: bilateral emmetropia (group EE), bilateral -0.5 D (group MM), and emmetropia and -0.5D (group EM).

After routine examinations 1 week or longer postoperatively, patients were asked for their preference in the fellow eye (Fig 1). Based on patient preference and the outcomes in the first eye, the targeted refraction for the fellow eye was determined to be emmetropia or -0.5 D. When the patient selected a different targeted refraction, the effects and risks of monovision were explained. For eyes targeting emmetropia, IOL powers for which the predicted refractions was negative number closest to 0.0 D were chosen to avoid postoperative hyperopia. For eyes targeting -0.5 D, the powers for which the predicted refraction was closest to -0.5 D were chosen. Consequently, the patients were divided into three groups based on the choice of the targeted refractions: bilateral emmetropia (group EE), bilateral -0.5 D (group MM), and emmetropia and -0.5 D (group EM). A minimal sample size was 19 patients in each group, which was required for detecting differences in the binocular uncorrected visual acuity (BUCVA) among the three groups with the significant level of 0.05 and detection power of 0.75 when the effect size was 0.4 (corresponding to large in Cohen’s classification).

### Examinations

Three months postoperatively, the BUCVAs were measured at the distances of 5.0, 1.0, 0.7, 0.5, and 0.3 meters and converted to the logarithm of the minimum angle of resolution (logMAR) for analysis. The manifest refraction spherical equivalents (MRSEs) also were recorded monocularly during examinations of the best-corrected visual acuity at 5 meters. Patients were questioned about their spectacle use.

The near stereopsis was measured at 16 inches using the Titmus stereoscopic test under photopic illumination. The numbers of answers for the three targets (fly, animal, and circle) without refractive correction were converted to seconds of arc for analysis, with 100 seconds of arc considered as the maximal limit of normal stereopsis [11,12].

Patient satisfaction and symptoms of photic phenomena were assessed through a questionnaire. Photic severities in glare, halos, starbursts, and low-contrast sensitivity were graded on a scale of 1 to 4, with 1 indicating no symptoms, 2 mild/rare, 3 moderate/occasional, and 4 severe. Satisfaction with the overall, distance, intermediate, and near vision also was graded on a scale of 1 to 4, with 1 indicating very unsatisfied, 2 unsatisfied, 3 satisfied, and 4 very satisfied. The mean of each score was calculated.

### Statistical analysis

Visual acuities, near stereopsis, patient satisfaction, and symptoms of phonic phenomena were compared among the three groups using the Kruskal-Wallis test following the Steel-Dwass multiple comparison test. The use of spectacles was evaluated using the Fisher’s exact test. P<0.05 was considered significant.

Although the postoperative MRSEs were targeted to emmetropia (0.0 D) or -0.5 D, it was difficult to achieve them with sufficiently small refractive errors. In recent assessments [13,14], refractive errors of 0.5 D or less were achievable in approximately 70% of eyes. So, a sub-analysis was conducted for exploring the effect of the -0.5 D targeted refractions. The EE and MM groups were re-classified based on the postoperative MRSE: patients with the MRSEs in the ranges of -0.25 to +0.25 D and -0.75 to -0.25 D were assigned to the EE and MM groups, respectively. The BUCVAs of the patients were compared using the Mann-Whitney test.

## Results

Groups EE, MM, and EM were comprised of 22, 21, and 21 patients, respectively. Table 1 shows the patient demographic data. There were no differences in the age and corneal astigmatism among the three groups (P = 0.072 and P = 0.43, respectively, the Kruskal-Wallis test). In the axial length and IOL power, the EE group differed significantly (P<0.0066 and P<0.017, respectively, the Steel-Dwass multiple comparison test) from the other groups. There were eyes of corneal astigmatism of 1.71 and 1.72 D in the MM and EM groups, respectively, the postoperative cylinders resulted in 1.25 and 0.0 D by adjusting the incision positions.

**Table 1:** Preoperative demographic data.

Group	EE	MM	EM	P value^+^
Targeted refractions	0.0 D x 2	-0.5 D x 2	0.0, -0.5 D	
No. of patients	22	21	21	
Age (years), range	69.6 ± 4.7, 59 to 82	65.0 ± 7.0, 51 to 79	68.2 ± 6.9, 55 to 81	0.072
Axial length (mm), range	24.0 ± 1.3*, 22.6 to 27.5	25.4 ± 1.8, 22.3 to 29.6	24.8 ± 1.5, 21.6 to 29.1	< 0.001
IOL power (D), range	20.4 ± 4.0*, 10.5 to 24.5	16.4 ± 5.4, 6.0 to 25.0	18.1 ± 4.5, 7.5 to 26.5	< 0.001
Corneal astigmatism (D), range	0.64 ± 0.37, 0.06 to 1.41	0.74 ± 0.39, 0.06 to 1.71	0.66 ± 0.38, 0.05 to 1.72	0.43

The data are expressed as the mean ± standard deviation.

^+^: Kruskal-Wallis test.

*: Significant difference between the MM and EM groups (P<0.017, the Steel-Dwass multiple comparison).

EE = bilateral emmetropia; MM = bilateral -0.5 D bilaterally; EM = monovision of emmetropia and -0.5 D; IOL = intraocular lens; D = diopters.

**Fig 2:**
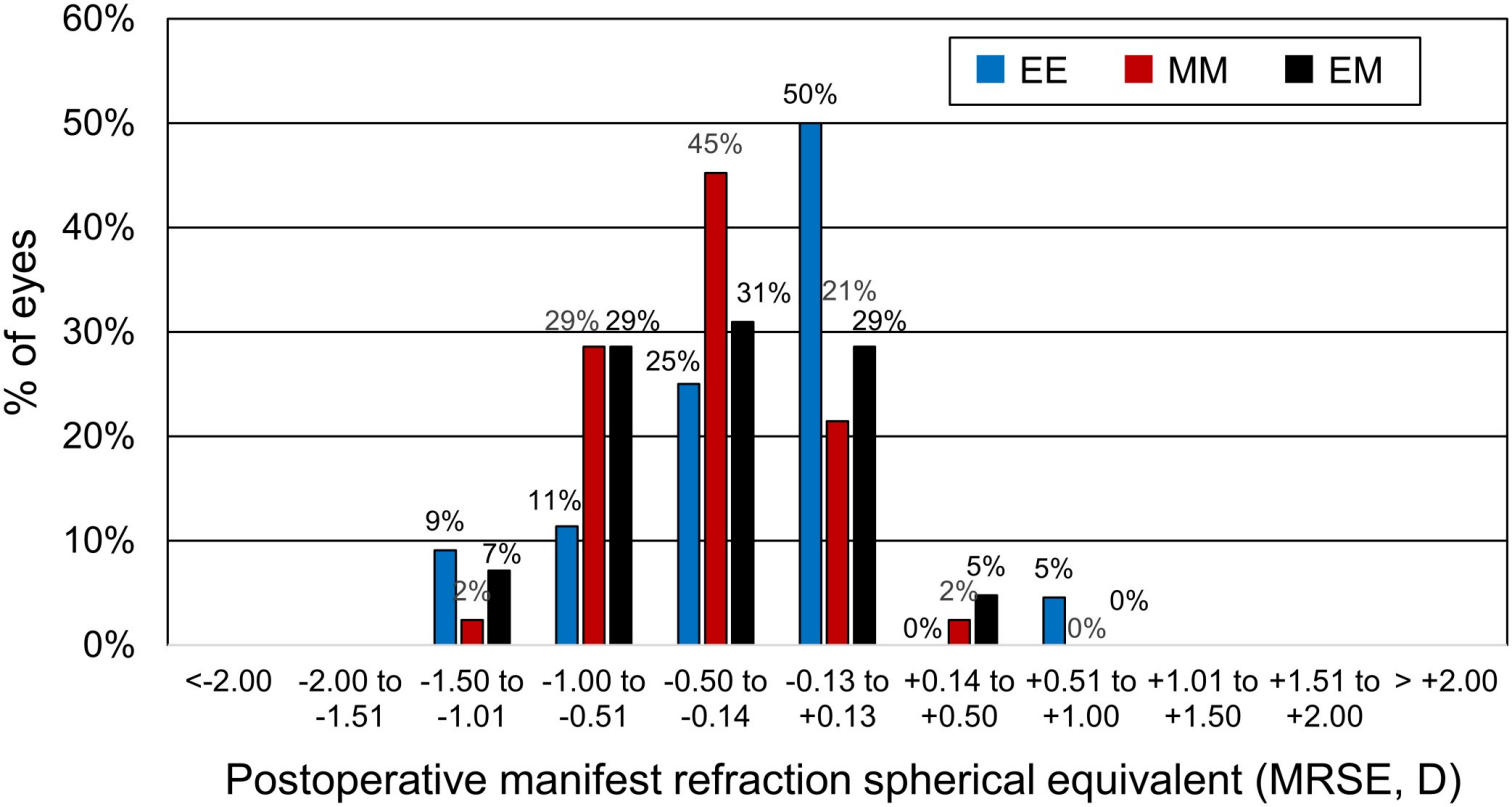
Histograms of postoperative manifest refraction spherical equivalent in three groups. Group EE: bilateral emmetropia, group MM: bilateral -0.5 diopters, and Group EM: monovision of emmetropia and -0.5 D.

Table 2 shows the postoperative MRSEs in the 3 groups. For the eyes targeted to emmetropia (N = 65) and -0.5 D (N = 63), the mean MRSEs were -0.23 D (standard deviation [SD]: 0.44 D, range: -1.50–1.00 D) and -0.51 D (SD: 0.37 D, range: -1.38–0.38 D), respectively. There was no significant difference between the EE group and the emmetropia targeted in the EM group (P = 0.44, t-test), while the MRSEs in the MM group were significantly (P = 0.047) higher than those in the -0.5-D-targeted eyes in the EM group. Fig 2 shows the histograms of the MRSEs [15]. There were peaks at emmetropia (-0.13 to 0.13 D) and the range of -0.50 to -0.13 D, while the EM resulted in broad peaks in the range of -1.00 to +0.13 D. Hyperopia were found in 2% to 5% in each group.

**Table 2:** Postoperative manifest refractions spherical equivalent (MRSE) in the three groups.

Group	EE (N = 44 eyes)	MM (N = 42 eyes)	EM (N = 21 eyes each)
MRSE (D), range			
Emmetropia targeted	-0.26 ± 0.48, -1.50 to 1.00	-	-0.17 ± 0.34, -0.75 to 0.50
-0.5 D targeted	-	-0.44 ± 0.35, -1.13 to 0.38	-0.64 ± 0.39, -1.38 to 0.00
Eyes within ± 0.3 D from target refractions			
Monocular, -0.3 to 0.3 D, eye	23 (52.3%)	-	12 (57.1%)
Monocular, -0.8 to -0.2 D, eye	-	26 (61.9%)	12 (57.1%)
Binocularly, patient	10 (45.5%)	8 (38.1%)	9 (42.9%)

The data are expressed as the mean ± standard deviation.

EE = bilateral emmetropia; MM = bilateral -0.5 D bilaterally; EM = monovision of emmetropia and -0.5 D; D = diopters.

As shown in Table 2, 42.9% to 61.9% of the monocular eyes were within ± 0.3 D of the targeted refractions; however, only 38% to 45% of patients achieved binocularity. The mean refractive cylinders in the EE, MM, and EM groups were 0.44, 0.50, and 0.45 D (SD: 0.42, 0.42, and 0.43 D), respectively, and there was no significant difference between the groups (P = 0.84, the Kruskal-Wallis test).

**Fig 3:**
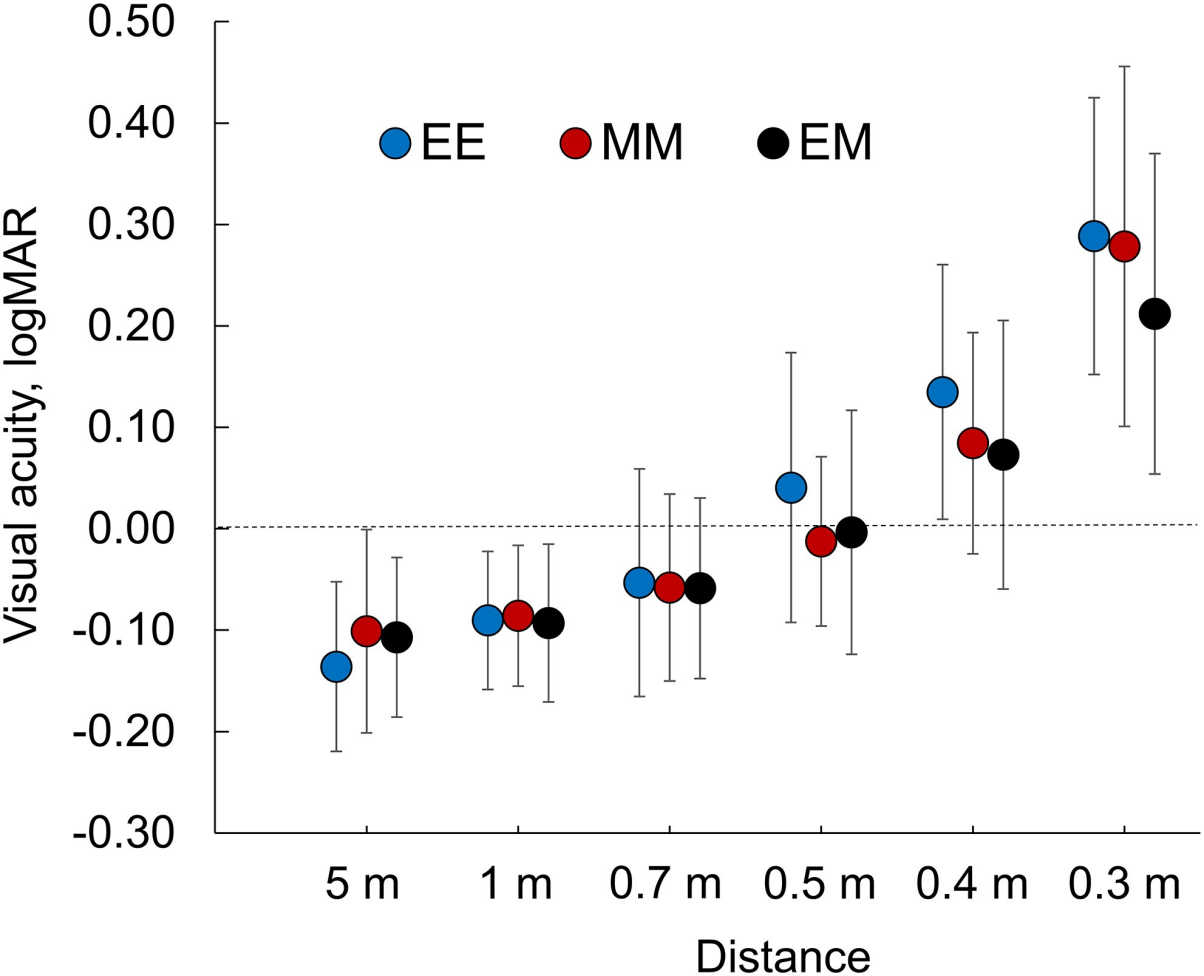
Binocular uncorrected visual acuities from 0.3 to 5.0 meter in three groups. Group EE: bilateral emmetropia, group MM: bilateral -0.5 diopters, and Group EM: monovision of emmetropia and -0.5 D. No significant differences are seen among the groups. logMAR = logarithm of the minimum angle of resolution.

**Fig 4:**
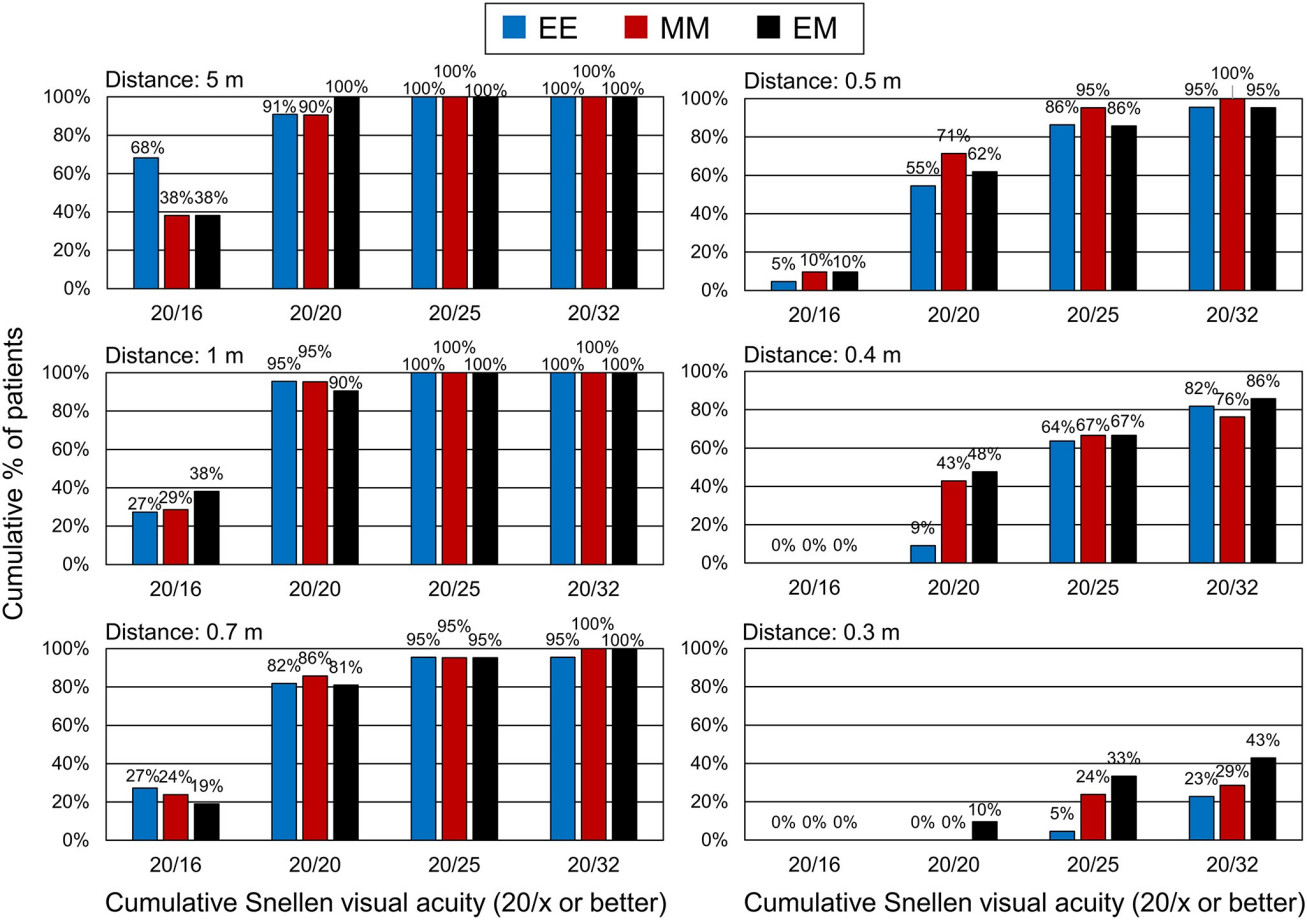
Histograms of binocular uncorrected visual acuities at distances of 5, 1, 0.7, 0.5, 0.4, and 0.3 meter in three groups. EE: bilateral emmetropia, group MM: bilateral -0.5 diopters, and Group EM: monovision of emmetropia and -0.5 D.

Fig 3 shows the BUCVAs from 0.3 to 5.0 meters. No differences were found among the three groups (P>0.23). The mean BUCVA was 0.0 logMAR (20/20 in Snellen notation) or better at 0.7 meter or longer in the EE group and 0.5 meters or longer in the MM and EM groups. The histogram of the BUCVA at each distance is shown in Fig 4 [15]. 20/20 or betters was achieved in more than 55% of patients, and it degraded at nearer distances drastically in the EE group.

In the EE, MM, and EM groups, 7(31.8%), 12(57.1%), and 11(52.4%) of patients did not use spectacles, a difference that did not reach significance (P = 0.13, Fisher’s exact test) among the groups.

The postoperative stereopsis were obtained in 22, 20, and 20 patients, and resulted in 53, 57, and 55 (SD: 23, 31, and 28) seconds of arc in the EE, MM, and EM groups, respectively. There was no significant difference (P = 0.70) among the groups. The rates in the normal range (100 seconds of arc or less) [11] were 95.5% (21 patients), 90.0% (18 patients), and 95.0% (19 patients), respectively.

Table 3 shows the scores of the photic symptoms and patient satisfaction in each group. Except for the satisfaction with closer distances such as when reading a book and using a smart phone, scores of 3 or 4 or higher were obtained. No significant (P>0.65) differences were found among the groups.

**Table 3:** Mean scores of photic symptom and patient satisfaction*.

Group	EE	MM	EM	P Value†
Photic symptom				
Glare	2.9 ± 0.9	3.0 ± 0.9	3.0 ± 0.9	0.70
Halo	3.0 ± 0.9	3.0 ± 1.0	3.0 ± 1.1	0.94
Starburst	2.9 ± 1.1	3.0 ± 0.9	3.0 ± 1.2	0.77
Waxy vision	3.5 ± 0.9	3.8 ± 0.4	3.5 ± 0.9	0.85
Satisfaction				
Far vision	3.3 ± 0.6	3.2 ± 0.7	3.0 ± 0.9	0.65
Intermediate vision	3.3 ± 0.5	3.2 ± 0.4	3.2 ± 0.7	0.88
Near vision	3.0 ± 0.5	3.0 ± 0.3	3.0 ± 0.9	1.00
Close vision	2.5 ± 0.7	2.5 ± 0.8	2.6 ± 0.9	0.96
Total	3.3 ± 0.5	3.3 ± 0.6	3.1 ± 0.9	0.73

The data are expressed as the mean ± standard deviation.

*The scores range from 1 (worst) to 4 (best).

^†^: the Kruskal-Wallis test.

**Fig 5:**
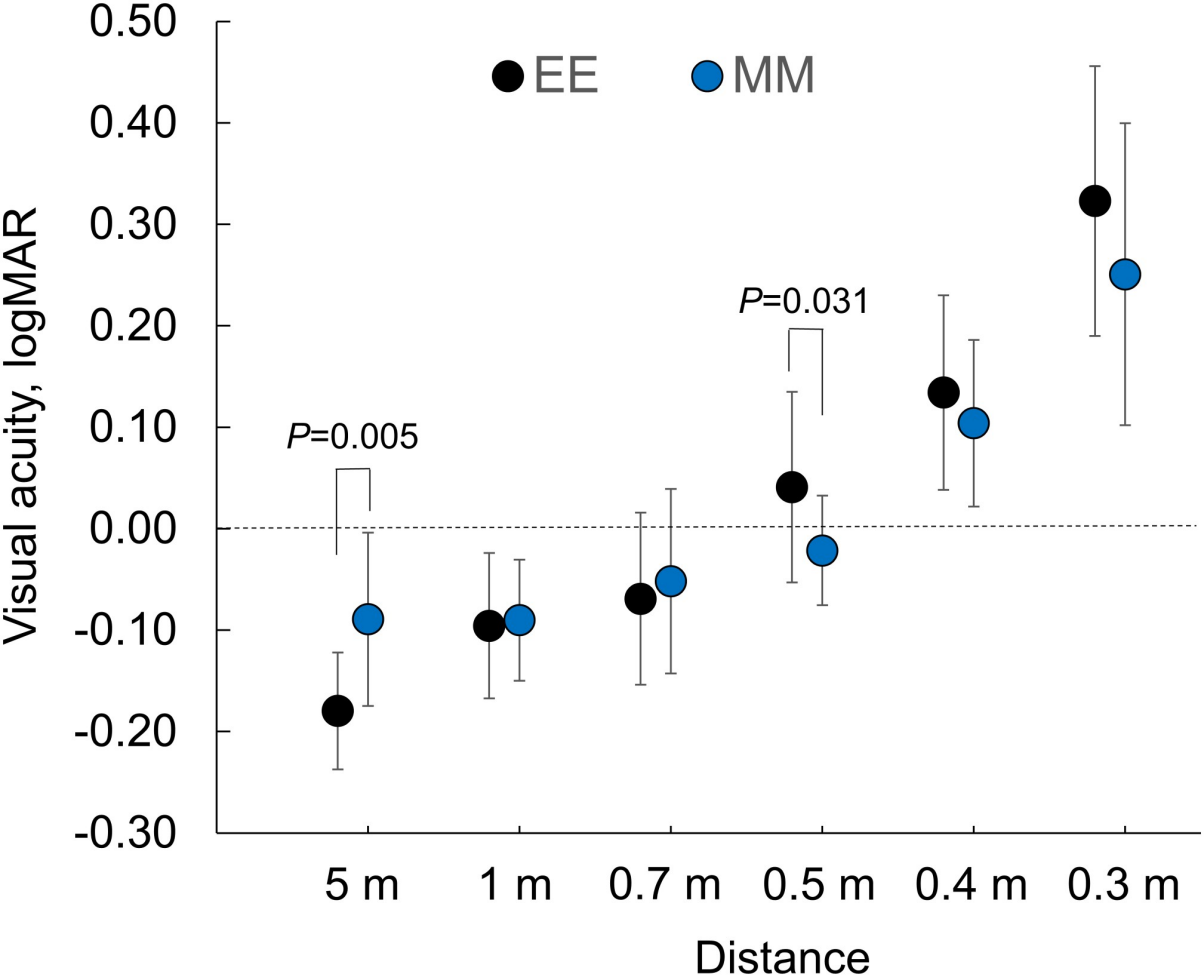
Binocular uncorrected visual acuities from 0.3 to 5.0 meter in the patients obtained the postoperative refractions in the ranges of -0.25 to +0.25 D (EE group) and -0.75 to -0.25 D (MM group) bilaterally. Significant differences were found at 5 m and 0.5 m (P = 0.005 and 0.031, the Mann-Whitney U-test). logMAR = logarithm of the minimum angle of resolution.

Based on the MRSE, 16 and 12 patients were assigned to the EE and MM groups, respectively. Fig 5 show the mean of the BUCVAs of the 2 groups The EE group showed a significantly better at 5 m (P = 0.005, Man-Whitney test) and the mean difference was 0.09 logMAR.

## Discussion

In the staged implantations of the IOL with the two targeted refractions, there were no differences in the BUCVA, stereopsis, photic symptoms, or patient satisfaction among the EE, MM, and EM groups. In a previous multicenter study, monovision targeting emmetropia and myopia (approximately -0.5 D) in 112 patients improved significantly in the BUCVAs at 0.4 and 0.7 m compared with the 299 patients in which bilateral emmetropia was targeted [5], as shown in Table 4. The mean MRSEs in the eyes targeted for -0.5 D resulted in -0.75 D, which could be most effective for monovision [6]. The current comparison between the EE and MM groups based on the MRSE showed that there was a significant difference at 0.5 m, as well. Thus, we speculated that the discrepancies with the previous study were due to the resultant MRSE in the eyes targeting for -0.5 D.

**Table 4:** Comparison of binocular uncorrected visual acuities in a previous study [5] with the current results.

Targeted refractions	Bilateral Emmetropia	Emmetropia and -0.5 D	P Values
Far			
Previous [5]	0.03 ± 0.09	0.04 ± 0.11	0.49
Current	-0.14 ± 0.08	-0.11 ± 0.08	0.14
0.7 meter			
Previous [5]	0.13 ± 0.16	0.09 ± 0.17	0.003
Current	-0.05 ± 0.11	-0.06 ± 0.09	0.95
0.4 meter			
Previous [5]	0.21 ± 0.16	0.17 ± 0.18	0.011
Current	0.13 ± 0.13	0.21 ± 0.16	0.13
MRSE, D			
Previous [5]	-0.30 ± 1.13	-0.21 ± 0.38 and -0.75 ± 0.52	
Current	-0.26 ± 0.48	-0.17 ± 0.34 and -0.64 ± 0.39	

The data are expressed as the mean ± standard deviation.

D = diopters; MRSE = manifest refraction spherical equivalent.

The EM group did not show the effect of monovision in the current results. It would be one of the factors that the subject size was not sufficiently large to distinguish between the EE and MM groups. The mean difference in the MRSE was 0.47 D, while Cochener reported that the best effect of monovision was obtained with the ZXR00 IOL when the MRSE difference was 0.75 D [6]. Further prospective evaluation is required.

Relatively large variations in the MRSEs were seen. The SDs of 0.34 to 0.48 D were comparable to differences in the targeted refraction (0.5 D), and only 38.1% to 45.5% of patients obtained binocular MRSEs within ± 0.3-D error. More accurate IOL power calculation is necessary to successfully achieve monovision with the EDOF IOLs. While current conventional IOL power calculations using optical biometry have been available, refractive errors within 0.5 D can be obtained in about 70% of eyes [13–16]. This result suggests that such refractive errors would be achievable binocularly in about 50% of patients, which agreed well with the current results. With the use of an advanced power calculation formula such as the Barrett Universal II, a refraction error within 0.25 D would be obtained in half of the eyes [16]. These findings suggested that targeting of emmetropia and -0.5 D was difficult to achieve, so that the effect of monovision would not be anticipated. We concluded that a larger difference in the targeting, such as emmetropia and a -0.75 D, would be practical.

There was no difference in the spectacle use, stereopsis, photic symptom, and patient satisfaction among the three groups. The spectacle use, photic symptom, and patient satisfaction were similar to those reported in the previous studies [6,7]. The stereopsis did not change when diffractive bifocal IOLs of different add powers were implanted bilaterally [12]. Targeting a -0.5 D was insufficient for modifying the BUCVA at intermediate and near distances, so that the effect should be less.

The current study had limitations. The sample sizes were not the same among the three groups. In the study design of staged implantation, it was not easy to adjust the sample sizes to accommodate the patients’ preferences. However, the current results showed the reality of targeting of a -0.5 D in the use of EDOF IOLs. Another limitation was the accuracy of the IOL power calculations. We used the SRK/T formula with biometric measurements together with spectrum-domain biometry. However, only half of the subjects had refractive errors within ± 0.3 D. Although the current biometry and IOL power calculation methodology has become advanced, the achieving 0.25 D differences would be still challenging. Furthermore, it was difficult to evaluate the effect of a -0.5-D refraction. Cochener [6] suggested in a retrospective analysis that targeting of -0.75 D would be better and more realistic to achieve. A prospective assessment of -0.75 D targeting is required to verify the effects.

In conclusion, targeting of -0.5 D in the use of an EDOF IOL resulted in similar outcomes with the emmetropic targeting due to the insufficient accuracy in the current IOL power calculations. Targeting a lower refraction such as -0.75 D would be more practical and effective for patients with bilateral implants.
